# A study of vertebra number in pigs confirms the association of vertnin and reveals additional QTL

**DOI:** 10.1186/s12863-015-0286-9

**Published:** 2015-10-30

**Authors:** Gary A. Rohrer, Dan J. Nonneman, Ralph T. Wiedmann, James F. Schneider

**Affiliations:** United States Department of Agriculture, Agricultural Research Service,, U.S. Meat Animal Research Center, Clay Center, NE 68933 USA

**Keywords:** Rib, Thoracic, Lumbar, Vertebra, Kyphosis, GWAS, Pig, Swine

## Abstract

**Background:**

Formation of the vertebral column is a critical developmental stage in mammals. The strict control of this process has resulted in little variation in number of vertebrae across mammalian species and no variation within most mammalian species. The pig is quite unique as considerable variation exists in number of thoracic vertebrae as well as number of lumbar vertebrae. At least two genes have been identified that affect number of vertebrae in pigs yet considerable genetic variation still exists. Therefore, a genome-wide association (GWA) analysis was conducted to identify additional genomic regions that affect this trait.

**Results:**

A total of 1883 animals were phenotyped for the number of ribs and thoracolumbar vertebrae as well as successfully genotyped with the Illumina Porcine SNP60 BeadChip. After data editing, 41,148 SNP markers were included in the GWA analysis. These animals were also phenotyped for kyphosis. Fifty-three 1 Mb windows each explained at least 1.0 % of the genomic variation for vertebrae counts while 16 regions were significant for kyphosis. Vertnin genotype significantly affected vertebral counts as well. The region with the largest effect for number of lumbar vertebrae and thoracolumbar vertebrae were located over the Hox B gene cluster and the largest association for thoracic vertebrae number was over the Hox A gene cluster. Genetic markers in significant regions accounted for approximately 50 % of the genomic variation. Less genomic variation for kyphosis was described by QTL regions and no region was associated with kyphosis and vertebra counts.

**Conclusions:**

The importance of the Hox gene families in vertebral development was highlighted as significant associations were detected over the A, B and C families. Further evaluation of these regions and characterization of variants within these genes will expand our knowledge on vertebral development using natural genetic variants segregating in commercial swine.

**Electronic supplementary material:**

The online version of this article (doi:10.1186/s12863-015-0286-9) contains supplementary material, which is available to authorized users.

## Background

Number of specific vertebrae in most mammalian species is quite conserved. The degree of conservation is location dependent with greater conservation towards the anterior end of the animal. For example, the number of cervical vertebrae in most mammalian species is fixed at seven whereas the number of caudal vertebrae among the 133 mammalian species observed ranged from 2 to 37 [1]. Variation in the number of thoracic and lumbar vertebrae across species does exist, but Narita and Kuratani [2] noted that the summation of thoracic and lumbar vertebrae (called thoracolumbar) was more conserved as most species were fixed at 19. Variation within species in thoracic, lumbar and thoracolumbar numbers has been observed in a limited number of mammalian species. The domestic pig is one such species where variation exists for vertebrae numbers. Surprisingly, a dramatic amount of variation exists in swine for a trait where most species exhibit no variation. Wild boars and Meishan pigs appear to be similar to most mammalian species with a thoracolumbar number of 19. However, commercial pigs of European origin typically range from 21 to 23 thoracolumbar vertebrae [3]. While it is often speculated that variation in pigs exists due to selection for increased body size, similar or greater changes in body size has occurred due to selection in other domesticated species (for example: cattle, sheep and dog) and yet variation within these species has not been widely reported.

Number of thoracolumbar vertebrae can affect carcass length, which is an economically important trait in pig production; however, vertebrae number only accounts for 10–15 % of the phenotypic variation in carcass length [3, 4]. Early reports indicated that pigs with more vertebrae suffered more lameness and structural problems [5, 6] as well as gave birth to more stillborn piglets [7]. These reports attempted to link disruption/modification of vertebral development with other abnormalities. Within the USMARC population a back curvature defect characterized as kyphosis has been observed and determined to have a genetic basis [8]. While this defect is not strictly associated with number of vertebrae, there is a tendency for increased incidence with greater number of ribs.

A number of genome scans have been conducted for carcass length and vertebra numbers in pigs. The first studies evaluated crosses between commercial breeds and either Meishan or Wild Boar pigs [9, 10]. Two major QTL were discovered in these studies and causative genetic variation has been identified in NR6A1 [11] and Vertnin [12]. Despite these discoveries, considerable unexplained genetic variation affecting thoracic, lumbar and thoracolumbar vertebra numbers still exists in commercial swine. Therefore, the objective of this study was to search for additional loci that affect vertebra numbers in a commercial composite swine population containing Duroc, Landrace and Yorkshire germplasm and determine if vertebra number is associated with animal performance or kyphosis.

## Methods

### Data collection

Care and handling of all animals included in this study was according to procedures outlined in *Guide for Care and Use of Agricultural Animals in Agricultural Research and Teaching* [13] and approved by the USMARC Animal Care and Use Committee. Animals were from a closed commercial-type composite population created by mating Duroc and Landrace boars to Yorkshire-Landrace composite females. Subsequently Duroc-sired animals were mated to Landrace-sired animals resulting in a population of ½ Landrace, ¼ Duroc and ¼ Yorkshire. Phenotyped animals were born in the fourth through eighth generations after composite formation (between May 2005 and November 2009). Animals were delivered to the USMARC abattoir and harvested using standard humane methods. The abattoir used electrical stunning followed by exsanguination under the supervision of USDA-FSIS inspectors. The age at slaughter ranged from 5 to 30 months. The number of ribs (RIB) and number of thoracolumbar (TLV) vertebrae were counted on the right side of the carcass. Number of ribs was considered the number of thoracic vertebrae and number of lumbar vertebrae (LVN) was computed by subtracting the number of ribs from the number of thoracolumbar vertebrae (LVN = TLV – RIB). Only animals with complete vertebra records were included in analyses.

Back curvature scores were also collected at the same time as vertebra counts based on the scoring system reported by Holl et al. [7]. The scoring was considered a continuous variable with values ranging from 0 (normal back curvature) to 3 (carcass exhibited severe kyphosis). All carcasses were scored by the same person.

Genomic DNA was extracted from tail tissue of all animals as described by Schneider et al. [14] and applied to Illumina Porcine SNP60 BeadChips [15] (Illumina Inc., San Diego, CA) per manufacturer’s instructions. Beadchips were read at USDA-ARS-BARC and data were interpreted with BeadStudio software (Illumina Inc; San Diego, CA). Markers with call rates < 80 %, minor allele frequencies of < 0.05 or significant deviation from Hardy-Weinberg equilibrium (*P* < 1.0 E −06) were eliminated from the analysis as well as markers not uniquely assigned to a position in build 10.2 of the porcine genome. Vertnin genotypes were determined for phenotyped animals by genotyping NV024 and NV090 [12] using the MassARRAY system (Agena BioSciences; San Diego, CA) based on manufacturer’s protocols. Pigs whose genotype was not determined with the MassARRAY system were imputed based on their genotype for MARC0038565 from the SNP60 BeadChip and linkage phase among these markers in the animal’s parents. A total of 726 phenotyped pigs as well as 188 parents of phenotyped pigs were genotyped for NV024 and NV090, and the remaining 1166 phenotyped animals had their genotypes imputed. The imputed genotypes of 96 animals were verified by PCR amplification of a fragment spanning the PRE-1 insertion site, reported as NV123 [12]. Vertnin genotypes were reported as the number of copies of the B allele for NV090.

The final data set included genotypic data from 41,148 markers. There were 1883 animals with vertebrae and genotypic data. For the back curvature, 2027 animals had both phenotypic and genotypic data available.

### Statistical analyses

Bayesian genome-wide association analyses were conducted using GenSel version 4.61R (www.animalgenome.org/community/projects/BIGS/). A covariate for vertnin genotype, expressed as number of copies of the B allele for NV090 [12], was fit in the model for all vertebra number traits. For kyphosis, only birth contemporary group was fitted as preliminary analyses indicated vertnin genotype did not affect this trait. Marker effects were fitted as additive effects based on the number of copies of the B allele (as defined by Illumina’s BeadStudio software).

A 1 Mb window analysis was conducted as described by Wolc et al. [16] and implemented in GenSel version 4.61R. This approach was used as linkage disequilibrium and marker density would tend to distribute the effect of a causative variant across multiple SNP markers in the analysis. Therefore, the genome was divided into non-overlapping 1 Mb windows and estimates of genetic variation attributed to each marker that had a non-zero effect within the window was summed for every 40^th^ iteration of the chain [16]. Under an infinitesimal model, each window would be expected to explain 0.04 % of the genomic variation. Thus, we concluded that windows explaining in excess of 10 fold the expectation (>0.4 %) should be considered a region of interest. Windows that accounted for more than 1 % of the genomic variation were considered significant, as all windows explaining >1.0 % genomic variation were determined to be highly significant (*P* < 0.001) after bootstrap analysis for other traits with similar heritability [17]. Within significant windows, the SNP marker with the largest estimated effect was reported.

Candidate genes within significant 1 Mb windows were selected after manually inspecting the regions using the UCSC Goldenpath genome browser (http://www.genome.ucsc.edu/), build 10.2 of the porcine genome. Annotated genes within QTL regions were considered candidates if they were involved in embryonic development or bone formation.

Association of number of vertebrae with performance was conducted using PROC GLM in SAS version 9.2 (Cary, NC). Weight and ultrasound estimated backfat thickness was recorded at 22 weeks of age on 3182 pigs and the model fit contemporary group and sex as fixed effects, age as a covariate and vertebra number analyzed as a covariate and as a categorical variable in separate analyses. Only one vertebra trait was fit at a time. Also, 1755 animals with vertebra counts had given birth to at least one litter. The percentage of stillborn piglets was analyzed with vertebra counts fitted similarly to weight and backfat analyses.

## Results

Descriptive statistics for the traits measured are presented in Table 1. Average values for RIB, LVN and TLV were 15.4, 6.1 and 21.6, respectively. The range observed for RIB was 14 to 17, LVN was 4 to 8 and TLV was 19 to 23. All SNP markers explained 16.1, 12.0, and 24.1 % of the total phenotypic variation of RIB, LVN, and TLV, respectively. The number of 1 Mb windows explaining more than 1 % of the genomic variation detected was 19 for RIB, 16 for LVN, and 18 for TLV. The percentage of genetic variation explained by these windows was 59 % for RIB, 48 % for LVN, and 52 % for TLV. The regression coefficients for vertnin genotype were significantly different from zero for RIB (−0.76), LVN (0.21), and TLV (−0.55).

**Table 1:** Descriptive statistics for phenotypic data analyzed in the study. Genomic variation is the amount of phenotypic variation associated with genotypic data and genomic heritability is the ratio of genomic to phenotypic variation

Trait	Mean	Range	Genomic Variation	Phenotypic variation	Genomic heritability
Thoracic vertebrae (RIB)	15.42	14 to 17	0.0203	0.1258	0.1610
Lumbar vertebrae (LVN)	6.12	4 to 8	0.0200	0.1656	0.1202
Thoracolumbar vertebrae (TLV)	21.55	19 to 23	0.0405	0.1677	0.2412
Kyphosis	0.33	0 to 3	0.0699	0.4222	0.1655

Table 2 displays the genomic regions significantly associated with at least one trait as well as additional associations of interest for these 1 MB windows. In total, loci affecting number of vertebra spanned 15 chromosomes and resided in 49 unique 1 Mb windows; however, eight pairs of adjacent 1 Mb windows were present and may actually represent only one causative genetic variant. If the adjacent regions were associated with the same vertebra trait, then the associations are likely due to a single causative gene based on strong linkage disequilibrium values (r^2^ > 0.99) between SNP markers with the largest estimated effects. This was evident for the adjacent regions of SSC 5: 70 and 71 Mb (RIB) and SSC 6:102 and 103 Mb (TLV). Chromosome 16 had three regions associated with LUM. Strong linkage disequilibrium was observed for the regions SSC 16:30 and 31 Mb, but less disequilibrium was observed between SSC 16:29 and 30 Mb (r^2^ = 0.56). The r^2^ values among the SNP markers with the largest estimated effects for the other four adjacent regions did not exceed 0.50.

**Table 2:** Results from GWAS for vertebral traits including chromosome, one megabase window and percent of genomic variation associated with the one megabase window for all significant associations (>1.0 %). Significant regions which were also suggestive (>0.4 %) for other traits are also listed. Potential candidate genes are presented in the last column

Chromosome	Mb	Thoracic variation	Lumbar variation	Thoracolumbar variation	Potential candidate gene symbol
4	114	1.11			MAB21L3
5	1	2.22			WNT7B
5	19		1.46		HOXC
5	70	1.80		0.40	TULP3
5	71	1.96			WNT5B
5	102	1.03			ALX1
6	81	2.35			MATN1
6	83	1.03			COL16A1
6	93		0.54	1.30	ARHGAP28
6	96			1.40	MYOM1
6	98			4.29	ADCYAP1
6	99	8.99		2.38	GATA6
6	102			1.13	ZNF521
6	103	0.59		2.68	ZNF521
6	146	2.86			LRP8
7	5			1.20	BMP6
7	54	1.04			ANKRD34C
7	103		1.20		VRTN
7	119			1.73	C14orf159
8	93			1.01	MAML3
8	98			1.06	PCDH10
9	14		2.30		TENM4
9	121		1.00		EZH2
9	124		6.03		TPK1
10	3		1.22		RGS18
11	25			1.49	AKAP11
12	19	1.28	1.25		MEOX1
12	24			10.26	HOXB
12	26		8.59	4.76	COL1A1
12	27			3.66	CHAD
12	34			6.21	MSI2
14	49	1.18			KREMEN1
14	75	1.38			SIRT1
14	80			1.23	UNC5B
15	30		1.18		CNTNAP5
16	18		4.80		CDH6
16	19			3.80	C5orf22
16	29		8.26		FGF10
16	30		1.08		FGF10
16	31		3.27		HCN1
16	45		1.20		RNF180
17	49		0.50	1.99	PTPRT
18	8	2.01			CLEC5A
18	48		2.36		FKBP14
18	50	17.35	2.70		HOXA
18	51	2.50			HOXA
18	54	2.80			RAMP3
X	41	4.72			CASK
X	140	1.58			MTMR1

There were no significant associations of any vertebra trait with first rib, last rib or last lumbar fat thickness (*P* > 0.50). Animals with more TLV grew faster (*P* < 0.03), but no other associations with growth were observed. Furthermore, there was no association between RIB, LVN or TLV of a sow and the number of stillborn piglets delivered in her first litter (*P* > 0.40).

The frequency of animals with some level of kyphosis was 24.7 %; however, most animals (17.4 %) were only mildly affected. The analysis of kyphosis indicated that genetic markers accounted for 16.6 % of the phenotypic variance. In total, 16 windows 1 Mb in size each explained more than 1 % of the genomic variation (Table 3). Cumulatively, these 16 windows explained 39.4 % of the genomic variation. These regions resided on 10 chromosomes and 13 unique regions separated by at least 10 Mb.

**Table 3:** Results from GWAS for kyphosis including chromosome, one megabase window and percent of genomic variation associated with the one megabase window for all significant associations (>1.0 %). Potential candidate genes are presented in the last column

Chromosome	Mb	Percent genomic variation	Potential candidate gene symbol
1	287	1.83	TNC
2	1	2.7	SHANK2
2	7	4.01	FLRT1
2	12	2.02	GLYAT
5	72	1.93	CECR2
5	73	2.28	CPNE8
6	60	1.07	CCDC27
6	105	1.55	
7	125	4.04	EMX2/VRK1
8	96	2.27	
13	145	1.87	ITGB5
15	143	1.39	
16	63	1.25	TENM2
X	5	2.27	
X	39	3.47	MID1IP1
X	136	5.45	SLITRK2

Additional file 1: Table S1 contains information on all 1 MB regions that exceeded 0.4 % of the genomic variation along with relevant data on the SNP within the window that had the largest estimated effect on the phenotype.

## Discussion

### Validation of Vertnin for vertebrae number

Variation in vertnin is clearly associated with the number of ribs and thoracolumbar vertebra in pigs. Our estimates of vertnin’s effect (0.76 ribs per copy of the mutant allele) on vertebra development concur with previous studies [12, 18]. The current study is the first genome wide association study that has fit variation in vertnin as a fixed effect. By accurately adjusting for variation in vertnin, we were able to detect additional loci affecting vertebra development in pigs that would have been masked without adjusting for vertnin (data not shown). In addition, the adjustment appeared to account for all phenotypic variation associated with this region for RIB and TLV. An association with SSC 7:103 Mb with LVN was detected. The most significant marker (DIAS0001088) exhibited a very low level of linkage disequilibrium (Additional file 2: Figure S1) with vertnin and was approximately 500 kb from vertnin, so it is possible a different gene is responsible for this association. Despite the large effect of vertnin, there are a number of other loci that affect the development of the vertebral column in pigs that can be exploited via selection to alter the number and type of vertebra in pigs. By utilizing this approach, 18 novel 1 Mb windows were discovered (based on QTLdb; http://www.animalgenome.org/cgi-bin/QTLdb/SS/index), and several other regions were only represented by QTL spanning 20 or more Mb.

### Comparison with other studies in swine for vertebrae number

Only four direct overlaps were observed among studies counting vertebrae. Ren and coworkers [19] found a QTL affecting both total vertebrae and thoracic vertebrae number on SSC 12 spanning 0.1-54.4 Mb in a population containing Duroc and Erhualian germplasm. Within this broad range, our study found seven different significant associations in five different 1 Mb windows (19, 24, 26, 27 and 34 Mb). All but one (19 Mb) significantly affected number of thoracolumbar vertebra. The window SSC 12:19 Mb affected both RIB and LUM while SSC 12:26 Mb was associated with LUM as well as TLV. Ren and coworkers [19] also reported a QTL spanning SSC 7:103 Mb for LUM. In a Duroc-Pietrain population, Edwards et al. [20] reported a QTL for number of ribs spanning SSC 7:54 Mb where we report a QTL for thoracic vertebra number. Harmegnies et al. [21] found QTL for rib number at SSC 18:24–52.2 Mb in a commercial population overlapping QTL for thoracic vertebra number at SSC 18:48–54 Mb in the present study. Surprisingly, the region of SSC 16:30–35 Mb was identified as controlling number of ribs in 2 different studies using commercial pigs [21, 22] but this region was associated with number of lumbar vertebrae in the current population.

Most published QTL that overlap these results measured carcass length rather than actual vertebra numbers. Several of these studies utilized F2 populations containing Chinese germplasm and overlap QTL detected in the current study on chromosomes 4 [23], 6 [24], 7 [25], 9 [24, 26], 14 [27], 17 [28], 18 [21] and X [9, 29]. Studies evaluating carcass length in commercial pigs confirmed QTL located on chromosome 6 [30–32], 12 [33] and 18 [33].

While carcass length is an economically important trait in pig production, vertebra numbers only account for a small proportion of the variation in carcass length [4]. Vertebra numbers have been associated with leaner carcasses [4] but data from our population was unable to substantiate this association (*P* > 0.50 for all analyses). Growth rate was associated (*P* < 0.03) with TLV but neither of the other two vertebra counts. Animals with more thoracolumbar vertebrae grew faster. Research reported nearly 50 years ago [5, 6] indicated that animals with more vertebrae suffered from lameness and movement problems. We do not monitor locomotion in our population, but one would presume lameness would result in reduced growth. If this presumption is correct, then animals with more thoracolumbar vertebra were not suffering from issues with mobility. A second production trait that has been associated with number of vertebrae is rate of stillbirths. Rees Evans [7] found that sows with more vertebrae had a higher incidence of stillborn piglets. Fredeen and Newman [34] contradicted the earlier report and our data also did not find any association with number of stillborn piglets and sow vertebra numbers (*P* > 0.40).

Four QTL regions were associated with more than one trait. The window of SSC 6:99 Mb was associated with coordinated changes in number of thoracic and thoracolumbar vertebrae while SSC 12:26 Mb was associated with coordinated changes in number of lumbar and thoracolumbar vertebrae numbers. Therefore these two regions increased the total number of vertebra but only affected one type of vertebra. Contrarily, SSC 18:50 Mb had a large effect on number of thoracic vertebrae and an opposite effect on number of lumbar vertebrae resulting in no significant change in number of thoracolumbar vertebra numbers. Finally, SSC 12:19 Mb had small effects on both thoracic and lumbar vertebra numbers with an undetectable effect on thoracolumbar vertebrae numbers. These associations were only marginally over the 1.0 % genomic variation threshold and the SNP markers with the largest effect on both traits were separated by 146 kb and had very different allele frequencies so the effects appear to be independent of each other.

To date, only two genes affecting vertebra numbers in pigs have been identified. Mikawa et al. [11] discovered a mutation in NR6A1 using crosses of Asian and European pigs , located on SSC 1:299 Mb; however, variation in this gene has been fixed in commercial (European descent) pigs based on genotypes and evidenced by a strong selective sweep [35]. No evidence of additional genetic variation in NR6A1 was found in the current population studied. Vertnin was discovered by the same group of scientists [12] and since its discovery it has been associated with vertebra numbers in multiple populations and within most germplasm [8,12,18,19]. Estimates of vertnin’s effect have been consistent at approximately 0.5 to 0.6 additional ribs (or thoracolumbar vertebrae) per copy of the mutant allele.

The genome wide association study discovered QTL for a surprisingly large percentage of the estimated genomic variation (48 to 59 %). The power to detect QTL in this study is quite high as it has the most phenotyped animals within a single population and utilized over 40,000 genetic markers. However, the genetic architecture of vertebral development should also be considered. Development of the vertebral column is a critically important process for survival, so it is highly conserved and strictly regulated [2]. Few species exhibit phenotypic variation for these traits and likely do not possess genetic variation. Therefore, it should not be surprising that the phenotypic variation observed in the pig may be due to a small set of genes. This was evident by our estimates of π in the Bayes Cπ analyses as estimates were > 0.999, indicating approximately 40 QTL should be expected. All of these facts together indicate that variation in vertebra numbers in pigs does not fit the infinitesimal model as well as it does a model where a few genes with moderate to large effects regulate this phenotype.

Further investigation of the QTL regions discovered in this study is necessary to determine the gene(s) associated with variation in vertebra numbers. This information will permit the use of marker-assisted selection decisions for producers wanting to change vertebra numbers in their populations as well as provide insight into the genetic mechanisms controlling this important developmental process. Determining the genes responsible for variation in vertebra numbers in pigs may also provide insight into why this developmental process is much less conserved in pigs than most other mammalian species.

### Identification of candidate genes for vertebrae number

Spatial cues which will result in segmental structures, such as vertebrae, result from the combinatorial expression of Hox genes within specific somites of the developing embryo [36, 37]. Manipulation of Hox genes can cause transformations of the vertebral column in mice [38, 39] and expression patterns of various Hox genes correlates with anatomical position across species [2]. Therefore, it shouldn’t be surprising that three of the QTL discovered are located where a cluster of Hox genes are located. The largest QTL for TLV (SSC 12:24) overlaps the *HoxB* cluster. This QTL tends to affect the number of total vertebrae or segments either independently of vertebra type or possibly by increasing number of lumbar vertebra (association detected at 24 Mb). The region associated with the most genomic variation for RIB was located over the *HoxA* (SSC 18:50 Mb) gene cluster. As previously noted this region appears to convert vertebrae from thoracic to lumbar or *vice versa*. Finally, the *HoxC* cluster (SSC 5:19 Mb) appears to have a small effect on number of lumbar vertebrae. Along with Hox genes, the WNT gene family also is a key regulator of embryonic development. Two RIB QTL colocate with the family members *WNT5B* (SSC 5:71.09 Mb) and *WNT7B* (SSC 5:0.66 Mb).

A few mutations have been studied in mice. One spontaneous recessive mutation caused split and/or fused ribs and was named rib-vertebrae [40]. Evaluation of this mutation implicated the Notch signaling pathway [41] and a discovery of a regulatory mutation in the gene *TBX6* was discovered [42]. While we did not find any associations near where *TBX6* should map, a family member, *TBX4*, does reside on SSC 12:38.23 Mb which is close to a QTL affecting TLV. Interestingly, *TBX4* is known to interact with *FGF10* which resides on SSC 16:30.24 Mb, which is central to LVN QTL on SSC16 at 29, 30 and 31 Mb. Another candidate gene associated with NOTCH signaling is MAML3 (SSC 8:92.37 Mb) located near a TLV QTL at SSC 8:93 Mb.

McPerron et al. [43] showed that knockout of *GDF11* resulted in anterior transformation of the vertebral column and increased the number of ribs in a dose dependent (additive) manner. *GDF11* in pigs is located on SSC 5:22.7 Mb only 3 Mb from a QTL associated with number of lumbar vertebrae, but the *HoxC* cluster is located directly in the QTL region and is a more likely candidate gene. *NODAL* is a TGF-beta superfamily member involved in early embryogenesis located at SSC 14:79.27 Mb, less than 1 Mb from a QTL for TLV (SSC 14:80) and a region of interest for RIB (Additional file 1: Table S1); thus, this gene may be responsible for the variation observed in the current study. Another candidate in this region at 80 Mb is the netrin-1 receptor *UNC5B*, which is a regulator of osteoclast differentiation [44]. Finally, teneurin-4 (*TENM4*) on SSC 9:14.33 Mb has been shown to be a critically important developmental gene necessary for the appropriate development of somites and thus the skeleton [45]. In addition, Lossie et al. [45] reported that mutations in the coding region of *TENM4* resulted in fusion of thoracic and/or lumbar vertebrae. *TENM4’s* location within a LVN QTL region and reported phenotypic effects of mutants in mice makes it a prime candidate for additional studies.

Several candidate genes involved in bone formation, osteoblast differentiation or chondrocyte differentiation include *ZNF521* on SSC 6:102.99 Mb [46], *BMP6* on SSC 7:5.16 Mb [47, 48], and *CHAD* on SSC 12:26.83 Mb [49] were all associated with TLV while *CLEC5A* on SSC 18:8.31 Mb and *RAMP3* on SSC 18:55.16 Mb were associated with RIB. While none of these genes have been reported to affect number of vertebra, their involvement in bone formation is intriguing and worthy of further investigation.

Several of these QTL overlap regions predicted to contain copy number variation based on analyses of SNP60 BeadChip data of the current population [50]. While many of the CNV in Wiedmann et al. [50] were predicted to segregate in only a few animals, QTL located on SSC 12:19 Mb (RIB and TLV), 16:19 Mb (TLV) and 16:29 Mb (LVN) were variable in over 20 families. However, predicted number of copies for specific animals by Wiedmann et al. [50] did not correlate well with phenotypic data adjusted for VRTN genotype.

### Genetic basis of Kyphosis

Kyphosis has been reported in commercial pigs. However, the kyphosis originally described by Holl et al. [7] within the USMARC and a Duroc-Landrace F2 population had not been previously reported. Lindholm-Perry et al. [51] conducted a genome scan on a subset of the animals used in the current study with a much lower marker density. They discovered several nominally significant associations in the USMARC and the Duroc-Landrace F2 population, but no associations were consistent across populations. Only a couple of the associations reported by Lindholm-Perry et al. [51] were near QTL reported in the current study. The QTL in the present study at SSC 5:72 and 73 Mb is near the association with marker 2928_3 (rs# 45434241), the QTL at SSC 6:60 Mb is only 5 Mb from PLOD1 which was associated with kyphosis in the linkage analysis and finally the QTL at SSC 6:105 Mb is near the microsatellite marker APR18. However, the largest QTL from the present study (SSC 2:7 Mb, 7:125 Mb and SSC X:136 Mb) were not detected by Lindholm-Perry et al. [51]. The difference in results can be explained by the nearly two-fold increase in phenotyped animals, a 200-fold increase in marker density and utilizing a Bayesian analysis.

While there were no direct overlapping associations for kyphosis and any vertebra trait, there were a number of QTL located within 2 Mb of each other. The large kyphosis QTL at SSC X:39 Mb (3.5 % genomic variation) was near the RIB QTL at SSC X:41 Mb (4.7 % genomic variation) while the kyphosis QTL at SSC 5:72–73 Mb (collectively accounting for 4.2 % of the genomic variation) was only 2 Mb away from a RIB QTL at SSC 5:70–71 Mb (collectively accounting for 3.8 % of the genomic variation). Whether these colocalizations are due to pleiotropic effects or merely linked QTL it may help explain the trend seen where for each additional rib, the frequency of mildly affected pigs increases by 3 %. The kyphosis QTL at SSC 6:105 Mb and 8:96 Mb were near TLV QTL, but both of these QTL had smaller estimated effects.

## Conclusion

Regulation of vertebral column development in pigs is controlled by numerous genes. In most species, genetic variation in this regulatory system is not tolerated. The pig is quite unique in the variability present and the amount of phenotypic variation displayed as a species. These results highlight the importance of the HOX gene families in embryonic development. Candidate genes in TGF-beta superfamily members were also detected. Further studies of vertebra numbers in pigs will provide insight into this developmental mechanism and provide natural genetic variants for future basic research. In addition, modification of vertebra numbers in commercial swine is possible and can be fortified by use of genetic markers.

## Additional files

Additional file 1: Table S1. All one megabase window associations explaining more than 0.4 % of the genomic variation for a trait, the most significant SNP and parameters associated with the most significant SNP such as location (build 10.2), effect, standard error, model frequency and allele frequency of the B allele. (DOCX 46 kb) Additional file 2: Figure S1. Linkage disequilibrium plot for SNP markers on chromosome 7 between 103 and 105 Mb. Figure created with Haploview 4.2 (http://www.broadinstitute.org/haploview/haploview). (DOCX 359 kb)

## Abbreviations

BMP6: bone morphogenetic protein 6
*CHAD*: chondroadherin
CLEL5A: C-type lectin domain family 5 member A
CNV: copy number variation
FASS: Federation of Animal Science Societies
FGF10: fibroblast growth factor 10
FSIS: Food Safety Inspection Service
GDF11: growth/differentiation factor 11
GWA: genome-wide association
HOX: Homeo box
LVN: number of lumbar vertebrae
Mb: megabase
NODAL: nodal homolog precursor
NR6A1: nuclear receptor subfamily 6 group A member 1
PLOD1: procollagen-lysine,2-oxoglutarate 5-dioxygenase 1
QTL: quantitative trait locus (loci)
RAMP3: receptor activity-modifying protein 3 precursor
RIB: number of ribs
SNP: single nucleotide polymorphism
SSC: sus scrofa
TBX6: T-box transcription factor 6
*TENM4*: teneurin-4
TLV: number of thoracolumbar
UNC5B: netrin receptor UNC5B precursor
USDA: United States Department of Agriculture
USMARC: U.S. Meat Animal Research Center
VRTN: vertnin
ZNF521: zinc finger protein 521
