# Supplementary material for: A study of vertebra number in pigs confirms the association of vertnin and reveals additional QTL
Source: BMC Genet. 2015 Oct 30;16:129. doi: 10.1186/s12863-015-0286-9 (PMC4628235; doi:10.1186/s12863-015-0286-9)
Supplement: Additional file 1: Table S1. — All one megabase window associations explaining more than 0.4 % of the genomic variation for a trait, the most significant SNP and parameters associated with the most significant SNP such as location (build 10.2), effect, standard error, model frequency and allele frequency of the B allele. (DOCX 46 kb) [file 12863_2015_286_MOESM1_ESM.docx]

**Additional file 1 Table S1** All one megabase window associations explaining more than 0.4 % of the genomic variation for a trait, the most significant SNP and parameters associated with the most significant SNP such as location (build 10.2) effect, standard error, model frequency and allele frequency of the B allele.

| Trait | SSC | Pos | %Var | SNP Name | Location | effect | se | ModFreq | f(B) |
| --- | --- | --- | --- | --- | --- | --- | --- | --- | --- |
| Lumbar | 1 | 96 | 0.91 | H3GA0002203 | 96833336 | -3.77E-03 | 2.28E-04 | 0.0603 | 0.212 |
| Lumbar | 1 | 97 | 0.71 | H3GA0002223 | 97978461 | 1.05E-03 | 7.54E-05 | 0.0211 | 0.612 |
| Thoracolumbar | 1 | 253 | 0.6 | ALGA0008231 | 253406113 | -4.25E-03 | 3.22E-04 | 0.0787 | 0.518 |
| Lumbar | 1 | 267 | 0.45 | ASGA0006294 | 267326452 | -2.55E-03 | 1.49E-04 | 0.0375 | 0.855 |
| Kyphosis | 1 | 276 | 0.51 | ALGA0008956 | 276338465 | -7.83E-03 | 6.43E-04 | 0.0931 | 0.381 |
| Kyphosis | 1 | 287 | 1.83 | ALGA0009617 | 287460902 | 1.28E-02 | 9.19E-04 | 0.1298 | 0.738 |
| Thoracic/Rib | 1 | 293 | 0.65 | MARC0105290 | 293916851 | 4.73E-03 | 2.72E-04 | 0.0862 | 0.78 |
| Kyphosis | 2 | 1 | 2.7 | DIAS0001270 | 1643104 | -4.27E-02 | 3.16E-03 | 0.4543 | 0.522 |
| Kyphosis | 2 | 7 | 4.01 | ASGA0008834 | 7058677 | 4.57E-02 | 2.43E-03 | 0.3356 | 0.1370 |
| Kyphosis | 2 | 12 | 2.02 | ALGA0119337 | 12304082 | 3.02E-02 | 2.36E-03 | 0.3208 | 0.386 |
| Thoracic/Rib | 2 | 105 | 0.73 | MARC0059442 | 105758550 | -6.12E-03 | 3.63E-04 | 0.1205 | 0.673 |
| Kyphosis | 2 | 124 | 0.4 | ASGA0011576 | 124525211 | -1.87E-03 | 1.89E-04 | 0.0261 | 0.527 |
| Thoracic/Rib | 3 | 5 | 0.58 | SIRI0001195 | 5864908 | -3.83E-03 | 2.48E-04 | 0.0758 | 0.68 |
| Thoracolumbar | 3 | 17 | 0.53 | M1GA0004133 | 17841090 | 6.76E-03 | 4.59E-04 | 0.1245 | 0.655 |
| Thoracic/Rib | 3 | 21 | 0.58 | ASGA0013855 | 21596232 | -4.70E-03 | 3.02E-04 | 0.0980 | 0.607 |
| Lumbar | 3 | 26 | 0.52 | MARC0101263 | 26425966 | -2.46E-03 | 1.25E-04 | 0.0313 | 0.901 |
| Thoracic/Rib | 3 | 27 | 0.71 | MARC0081581 | 27964942 | -2.22E-03 | 1.51E-04 | 0.0485 | 0.449 |
| Thoracolumbar | 3 | 117 | 0.54 | ALGA0020906 | 117127099 | -5.62E-03 | 3.99E-04 | 0.1033 | 0.378 |
| Kyphosis | 4 | 12 | 0.57 | H3GA0011992 | 12332932 | 6.71E-03 | 5.33E-04 | 0.0774 | 0.307 |
| Kyphosis | 4 | 16 | 0.4 | DIAS0000481 | 16391213 | 1.66E-03 | 1.55E-04 | 0.0212 | 0.619 |
| Kyphosis | 4 | 61 | 0.74 | ASGA0096955 | 61457866 | -8.35E-03 | 5.62E-04 | 0.0753 | 0.199 |
| Kyphosis | 4 | 62 | 0.42 | ALGA0025247 | 62303926 | 2.14E-03 | 1.74E-04 | 0.0248 | 0.712 |
| Kyphosis | 4 | 71 | 0.43 | MARC0054025 | 71995864 | -5.68E-03 | 4.68E-04 | 0.0647 | 0.395 |
| Thoracic/Rib | 4 | 114 | 1.11 | H3GA0013916 | 114089519 | -1.27E-02 | 6.12E-04 | 0.1784 | 0.865 |
| Kyphosis | 4 | 116 | 0.77 | H3GA0014008 | 116430410 | 1.12E-02 | 9.44E-04 | 0.1375 | 0.39 |
| Thoracic/Rib | 5 | 1 | 2.22 | ASGA0106073 | 1046915 | 3.17E-02 | 1.27E-03 | 0.3691 | 0.914 |
| Lumbar | 5 | 19 | 1.46 | ASGA0085630 | 19332280 | 1.17E-02 | 6.05E-04 | 0.1596 | 0.84 |
| Lumbar | 5 | 20 | 0.99 | DIAS0002299 | 20616342 | -5.79E-03 | 3.79E-04 | 0.1037 | 0.337 |
| Thoracic/Rib | 5 | 23 | 0.47 | MARC0081195 | 23777569 | 4.14E-03 | 2.76E-04 | 0.0855 | 0.365 |
| Thoracolumbar | 5 | 69 | 0.98 | M1GA0007918 | 69333042 | 1.53E-02 | 7.91E-04 | 0.2068 | 0.854 |
| Thoracic/Rib | 5 | 70 | 1.8 | ALGA0032638 | 70775116 | -7.68E-03 | 4.78E-04 | 0.1481 | 0.345 |
| Thoracolumbar | 5 | 70 | 0.4 | MARC0071620 | 70181543 | 3.72E-03 | 2.14E-04 | 0.0536 | 0.858 |
| Thoracic/Rib | 5 | 71 | 1.96 | H3GA0016710 | 71310644 | 1.15E-02 | 7.07E-04 | 0.2183 | 0.653 |
| Kyphosis | 5 | 72 | 1.93 | ALGA0032754 | 72528496 | 2.76E-02 | 1.87E-03 | 0.2584 | 0.735 |
| Trait | SSC | Pos | %Var | SNP Name | Location | effect | se | ModFreq | f(B) |
| Kyphosis | 5 | 73 | 2.28 | ASGA0088507 | 73096221 | -3.44E-02 | 2.57E-03 | 0.352 | 0.56 |
| Kyphosis | 5 | 94 | 0.98 | ASGA0026725 | 94030528 | 1.63E-02 | 1.25E-03 | 0.1804 | 0.672 |
| Thoracic/Rib | 5 | 102 | 1.03 | ALGA0033839 | 102574608 | 5.77E-03 | 3.46E-04 | 0.1082 | 0.75 |
| Thoracolumbar | 5 | 106 | 0.43 | ASGA0027165 | 106682107 | 4.75E-03 | 2.62E-04 | 0.0651 | 0.12 |
| Kyphosis | 6 | 60 | 1.07 | ASGA0103561 | 60202083 | 1.77E-02 | 1.29E-03 | 0.1743 | 0.219 |
| Thoracic/Rib | 6 | 81 | 2.35 | ASGA0028790 | 81833437 | -1.62E-02 | 9.39E-04 | 0.2927 | 0.625 |
| Thoracic/Rib | 6 | 82 | 0.41 | MARC0006639 | 82245852 | 1.53E-03 | 9.80E-05 | 0.0308 | 0.376 |
| Thoracic/Rib | 6 | 83 | 1.03 | ALGA0035933 | 83877858 | -6.20E-03 | 2.44E-04 | 0.0699 | 0.925 |
| Lumbar | 6 | 93 | 0.54 | ASGA0084308 | 93699966 | 2.95E-03 | 1.99E-04 | 0.0548 | 0.399 |
| Thoracolumbar | 6 | 93 | 1.3 | ASGA0084308 | 93699966 | 1.14E-02 | 4.90E-04 | 0.1404 | 0.399 |
| Lumbar | 6 | 94 | 0.4 | ASGA0085561 | 94295946 | -2.52E-03 | 1.75E-04 | 0.0461 | 0.458 |
| Thoracolumbar | 6 | 96 | 1.4 | MARC0051654 | 96559074 | -2.91E-02 | 1.15E-03 | 0.2498 | 0.907 |
| Thoracolumbar | 6 | 98 | 4.29 | ALGA0036710 | 98518507 | -4.46E-02 | 1.57E-03 | 0.3688 | 0.813 |
| Thoracic/Rib | 6 | 99 | 8.99 | ASGA0029138 | 99847064 | -5.40E-02 | 2.51E-03 | 0.7724 | 0.543 |
| Thoracolumbar | 6 | 99 | 2.38 | ASGA0029138 | 99847064 | -1.90E-02 | 6.89E-04 | 0.192 | 0.543 |
| Thoracic/Rib | 6 | 100 | 0.56 | ASGA0098402 | 100318405 | 1.45E-03 | 7.77E-05 | 0.024 | 0.58 |
| Thoracolumbar | 6 | 102 | 1.13 | ASGA0029171 | 102763903 | 9.46E-03 | 3.77E-04 | 0.1021 | 0.397 |
| Thoracic/Rib | 6 | 103 | 0.59 | ASGA0029176 | 103223108 | 1.84E-03 | 8.73E-05 | 0.0267 | 0.784 |
| Thoracolumbar | 6 | 103 | 2.68 | MARC0040275 | 103322842 | 2.13E-02 | 8.28E-04 | 0.2232 | 0.398 |
| Lumbar | 6 | 104 | 0.47 | ALGA00366380 | 104960363 | -1.04E-03 | 7.11E-05 | 0.0199 | 0.604 |
| Kyphosis | 6 | 105 | 1.55 | ALGA0120579 | 105960772 | -2.15E-02 | 1.55E-03 | 0.2173 | 0.704 |
| Thoracolumbar | 6 | 105 | 0.4 | ASGA0029220 | 105589839 | -3.60E-03 | 1.86E-04 | 0.0524 | 0.452 |
| Kyphosis | 6 | 107 | 0.99 | MARC0020494 | 107035144 | -1.05E-02 | 7.82E-04 | 0.1095 | 0.726 |
| Thoracic/Rib | 6 | 142 | 0.48 | MARC0026081 | 142661416 | 3.35E-03 | 2.23E-04 | 0.0691 | 0.527 |
| Thoracic/Rib | 6 | 146 | 2.86 | ASGA0030060 | 146548402 | -2.23E-02 | 1.35E-03 | 0.412 | 0.328 |
| Thoracolumbar | 7 | 5 | 1.2 | H3GA0019664 | 5171639 | -1.57E-02 | 9.44E-04 | 0.2406 | 0.75 |
| Thoracolumbar | 7 | 20 | 0.43 | ASGA0031604 | 20097822 | -2.18E-03 | 1.58E-04 | 0.0405 | 0.353 |
| Thoracolumbar | 7 | 31 | 0.41 | ALGA0039930 | 31270305 | 4.06E-03 | 3.00E-04 | 0.0752 | 0.363 |
| Thoracolumbar | 7 | 41 | 0.8 | H3GA0021152 | 41569941 | 7.00E-03 | 4.66E-04 | 0.1208 | 0.453 |
| Kyphosis | 7 | 53 | 0.91 | ALGA0041544 | 53357762 | -1.62E-02 | 1.06E-03 | 0.1413 | 0.867 |
| Thoracic/Rib | 7 | 54 | 1.04 | INRA0025809 | 54417391 | -8.11E-03 | 4.66E-04 | 0.1429 | 0.728 |
| Thoracic/Rib | 7 | 69 | 0.51 | H3GA0021998 | 69284159 | -3.57E-03 | 2.40E-04 | 0.0747 | 0.484 |
| Kyphosis | 7 | 101 | 0.44 | M1GA0010637 | 101888771 | -3.84E-03 | 3.20E-04 | 0.0437 | 0.761 |
| Lumbar | 7 | 103 | 1.2 | H3GA0022664 | 103910821 | -1.54E-03 | 1.08E-03 | 0.0306 | 0.45 |
| Thoracolumbar | 7 | 113 | 0.42 | MARC0059131 | 113908824 | 6.82E-03 | 4.21E-04 | 0.1097 | 0.721 |
| Thoracolumbar | 7 | 119 | 1.73 | MARC0049988 | 119379640 | -2.68E-02 | 1.64E-03 | 0.4112 | 0.685 |
| Kyphosis | 7 | 125 | 4.04 | H3GA0023474 | 125047630 | -5.80E-02 | 4.30E-03 | 0.6073 | 0.541 |
| Thoracic/Rib | 8 | 6 | 0.57 | MARC0011781 | 6756275 | 3.41E-03 | 1.73E-04 | 0.0506 | 0.11 |
| Lumbar | 8 | 89 | 0.53 | MARC0095252 | 89162152 | 4.59E-03 | 2.19E-04 | 0.0577 | 0.898 |
| Thoracolumbar | 8 | 89 | 0.78 | MARC0095252 | 89162152 | 1.20E-02 | 5.68E-04 | 0.1406 | 0.898 |

| Trait | SSC | Pos | %Var | SNP Name | Location | effect | se | ModFreq | f(B) |
| --- | --- | --- | --- | --- | --- | --- | --- | --- | --- |
| Thoracolumbar | 8 | 93 | 1.01 | ASGA0039327 | 93900503 | -1.25E-02 | 6.57E-04 | 0.1684 | 0.841 |
| Kyphosis | 8 | 96 | 2.27 | INRA0030111 | 96557136 | -4.67E-02 | 2.63E-03 | 0.3486 | 0.878 |
| Thoracolumbar | 8 | 98 | 1.06 | ALGA0048677 | 98667343 | 1.97E-02 | 1.05E-03 | 0.2647 | 0.173 |
| Kyphosis | 8 | 125 | 0.62 | H3GA0056457 | 125450903 | 1.35E-02 | 9.06E-04 | 0.1286 | 0.821 |
| Lumbar | 8 | 130 | 0.47 | ALGA0049463 | 130653806 | 2.63E-03 | 1.59E-04 | 0.0446 | 0.21 |
| Lumbar | 9 | 14 | 2.3 | MARC0070952 | 14861213 | 1.47E-02 | 9.50E-04 | 0.2429 | 0.618 |
| Thoracolumbar | 9 | 18 | 0.41 | ALGA0120009 | 18939110 | 2.28E-03 | 1.83E-04 | 0.0458 | 0.35 |
| Lumbar | 9 | 22 | 0.58 | MARC0048836 | 22280834 | 4.53E-03 | 3.17E-04 | 0.082 | 0.577 |
| Lumbar | 9 | 39 | 0.9 | DRGA0009334 | 39034059 | -2.01E-03 | 1.27E-04 | 0.0365 | 0.357 |
| Thoracolumbar | 9 | 39 | 0.81 | DRGA0009334 | 39034059 | -6.42E-03 | 4.16E-04 | 0.1105 | 0.357 |
| Lumbar | 9 | 41 | 0.86 | DRGA0009996 | 41344444 | -2.42E-03 | 1.48E-04 | 0.0429 | 0.355 |
| Lumbar | 9 | 121 | 1 | ASGA0044277 | 121149973 | 3.62E-03 | 2.26E-04 | 0.0617 | 0.401 |
| Lumbar | 9 | 124 | 6.03 | ALGA0054715 | 124513811 | -2.23E-02 | 1.22E-03 | 0.3372 | 0.284 |
| Kyphosis | 9 | 144 | 0.64 | H3GA0028483 | 144214338 | 1.27E-02 | 9.40E-04 | 0.1329 | 0.249 |
| Lumbar | 10 | 3 | 1.22 | H3GA0028940 | 3301458 | -1.16E-02 | 5.49E-04 | 0.1434 | 0.883 |
| Lumbar | 10 | 9 | 0.81 | DRGA0010240 | 9888697 | 3.08E-03 | 2.09E-04 | 0.0592 | 0.599 |
| Thoracic/Rib | 10 | 10 | 0.89 | MARC0022071 | 10036397 | 4.98E-03 | 2.98E-04 | 0.0914 | 0.22 |
| Lumbar | 10 | 15 | 0.55 | H3GA0029351 | 15992149 | -7.01E-04 | 5.08E-05 | 0.0144 | 0.61 |
| Kyphosis | 10 | 39 | 0.7 | MARC0079978 | 39272859 | 4.13E-03 | 3.80E-04 | 0.05226 | 0.578 |
| Thoracolumbar | 11 | 25 | 1.49 | M1GA0026237 | 25783002 | 9.06E-03 | 6.24E-04 | 0.1576 | 0.561 |
| Thoracic/Rib | 11 | 31 | 0.54 | DRGA0011059 | 31515288 | 5.72E-03 | 2.94E-04 | 0.0865 | 0.844 |
| Kyphosis | 11 | 54 | 0.78 | MARC0115079 | 54595809 | -5.61E-03 | 5.06E-04 | 0.0698 | 0.532 |
| Kyphosis | 11 | 66 | 0.49 | ASGA0051058 | 66142455 | 5.86E-03 | 5.24E-04 | 0.0737 | 0.482 |
| Thoracolumbar | 11 | 76 | 0.49 | ASGA0051657 | 76165093 | 1.20E-02 | 5.92E-04 | 0.1499 | 0.101 |
| Thoracolumbar | 12 | 10 | 0.62 | ALGA0064900 | 10698900 | -5.48E-03 | 4.04E-04 | 0.0996 | 0.526 |
| Lumbar | 12 | 19 | 1.25 | H3GA0033824 | 19499414 | 8.27E-03 | 5.09E-04 | 0.1281 | 0.771 |
| Thoracic/Rib | 12 | 19 | 1.28 | H3GA0033801 | 19352812 | 1.03E-02 | 6.90E-04 | 0.2069 | 0.585 |
| Thoracolumbar | 12 | 24 | 10.26 | MARC0013292 | 24901249 | -7.40E-03 | 3.20E-03 | 0.8077 | 0.529 |
| Lumbar | 12 | 26 | 8.59 | DIAS0000635 | 26289830 | -4.86E-02 | 2.54E-03 | 0.7069 | 0.443 |
| Thoracolumbar | 12 | 26 | 4.76 | H3GA0033984 | 26969612 | 2.78E-02 | 1.28E-03 | 0.317 | 0.653 |
| Thoracolumbar | 12 | 27 | 3.66 | ALGA0065768 | 27013251 | 2.91E-02 | 1.43E-03 | 0.3599 | 0.418 |
| Thoracolumbar | 12 | 34 | 6.21 | M1GA0016584 | 34552177 | 8.33E-02 | 2.99E-03 | 0.7523 | 0.15 |
| Lumbar | 12 | 37 | 0.52 | ALGA0066234 | 37268596 | -1.53E-03 | 9.64E-05 | 0.0263 | 0.374 |
| Thoracolumbar | 12 | 37 | 0.99 | MARC0021670 | 37005248 | 6.20E-03 | 3.65E-04 | 0.0928 | 0.464 |
| Thoracic/Rib | 12 | 51 | 0.72 | ASGA0054989 | 51375066 | -7.37E-03 | 3.93E-04 | 0.1178 | 0.834 |
| Thoracolumbar | 12 | 51 | 0.82 | ASGA0054989 | 51375066 | -1.53E-02 | 8.64E-04 | 0.2111 | 0.834 |
| Kyphosis | 13 | 145 | 1.87 | ALGA0072090 | 145096895 | 3.52E-02 | 2.20E-03 | 0.2934 | 0.163 |
| Kyphosis | 13 | 187 | 0.58 | CASI0007727 | 187574046 | 5.40E-03 | 4.08E-04 | 0.0569 | 0.241 |
| Kyphosis | 13 | 194 | 0.86 | DRGA0013310 | 194386412 | -8.88E-03 | 7.27E-04 | 0.1046 | 0.522 |
| Thoracic/Rib | 14 | 48 | 0.86 | MARC0021603 | 48171457 | 6.92E-04 | 4.57E-05 | 0.0135 | 0.469 |

| Trait | SSC | Pos | %Var | SNP Name | Location | effect | se | ModFreq | f(B) |
| --- | --- | --- | --- | --- | --- | --- | --- | --- | --- |
| Thoracic/Rib | 14 | 49 | 1.18 | DRGA0013810 | 49925754 | -9.21E-04 | 5.90E-05 | 0.0172 | 0.531 |
| Thoracic/Rib | 14 | 50 | 0.64 | MARC0006678 | 50044442 | -6.64E-04 | 4.34E-05 | 0.0131 | 0.531 |
| Thoracic/Rib | 14 | 75 | 1.38 | DRGA0014070 | 75381575 | 6.67E-03 | 3.27E-04 | 0.1019 | 0.325 |
| Thoracic/Rib | 14 | 77 | 0.92 | ALGA0079525 | 77679226 | 4.88E-03 | 2.29E-04 | 0.0684 | 0.273 |
| Kyphosis | 14 | 78 | 0.5 | H3GA0041315 | 78194962 | 7.89E-03 | 6.33E-04 | 0.0878 | 0.6540 |
| Thoracic/Rib | 14 | 78 | 0.75 | INRA0045518 | 78271405 | -5.17E-03 | 2.44E-04 | 0.0725 | 0.727 |
| Thoracic/Rib | 14 | 79 | 0.52 | ALGA0078815 | 79501142 | -3.12E-03 | 1.85E-04 | 0.0587 | 0.527 |
| Thoracolumbar | 14 | 80 | 1.23 | ASGA0064482 | 80166032 | -8.32E-03 | 5.59E-04 | 0.142 | 0.49 |
| Thoracic/Rib | 14 | 89 | 0.85 | MARC0029597 | 89073094 | -2.68E-03 | 1.39E-04 | 0.0437 | 0.786 |
| Thoracic/Rib | 14 | 90 | 0.69 | ALGA0079375 | 90925232 | 1.25E-03 | 7.08E-05 | 0.0222 | 0.247 |
| Thoracolumbar | 14 | 119 | 0.5 | CASI0007888 | 119514048 | 6.77E-03 | 4.82E-04 | 0.1195 | 0.601 |
| Thoracic/Rib | 14 | 121 | 0.44 | H3GA0042070 | 121721589 | 1.38E-03 | 6.75E-05 | 0.0199 | 0.106 |
| Thoracic/Rib | 14 | 122 | 0.57 | INRA0046735 | 122482603 | 1.44E-03 | 8.39E-05 | 0.0262 | 0.21 |
| Thoracolumbar | 14 | 122 | 0.63 | INRA0046735 | 122482603 | 4.15E-03 | 2.55E-04 | 0.0643 | 0.21 |
| Lumbar | 15 | 30 | 1.18 | ASGA0098906 | 30264712 | 7.56E-03 | 4.48E-04 | 0.1308 | 0.668 |
| Thoracolumbar | 15 | 52 | 0.43 | MARC0001306 | 52840350 | -1.98E-03 | 1.44E-04 | 0.0354 | 0.23 |
| Kyphosis | 15 | 143 | 1.39 | ASGA0071235 | 143284148 | 9.24E-03 | 8.04E-04 | 0.1144 | 0.501 |
| Lumbar | 16 | 18 | 4.8 | H3GA0046086 | 18134235 | -3.09E-02 | 1.54E-03 | 0.4623 | 0.363 |
| Thoracolumbar | 16 | 19 | 3.8 | ASGA0072459 | 19022068 | -6.76E-02 | 3.04E-03 | 0.7667 | 0.148 |
| Lumbar | 16 | 29 | 8.26 | H3GA0046366 | 29952113 | 1.95E-02 | 8.01E-04 | 0.1736 | 0.289 |
| Lumbar | 16 | 30 | 1.08 | MARC0032144 | 30669418 | -2.87E-03 | 1.48E-04 | 0.0314 | 0.293 |
| Lumbar | 16 | 31 | 3.27 | ALGA0090017 | 31103652 | 6.86E-03 | 3.61E-04 | 0.0729 | 0.707 |
| Thoracolumbar | 16 | 42 | 0.62 | ASGA0073187 | 42826393 | 4.28E-03 | 3.17E-04 | 0.0797 | 0.497 |
| Lumbar | 16 | 45 | 1.2 | ALGA0090526 | 45678949 | 3.60E-03 | 2.18E-04 | 0.065 | 0.554 |
| Kyphosis | 16 | 63 | 1.25 | ASGA0073682 | 63799514 | -2.07E-02 | 1.64E-03 | 0.2333 | 0.622 |
| Thoracic/Rib | 17 | 29 | 0.42 | ASGA0075923 | 29886296 | -1.70E-03 | 1.13E-04 | 0.0358 | 0.273 |
| Lumbar | 17 | 49 | 0.5 | ASGA0091572 | 49835922 | 2.96E-03 | 2.06E-04 | 0.0587 | 0.55 |
| Thoracolumbar | 17 | 49 | 1.99 | ASGA0091572 | 49835922 | 2.60E-02 | 1.74E-03 | 0.4535 | 0.55 |
| Thoracic/Rib | 18 | 8 | 2.01 | MARC0112998 | 8380737 | 5.95E-03 | 3.45E-04 | 0.1037 | 0.309 |
| Lumbar | 18 | 48 | 2.36 | ALGA0098387 | 48388657 | -1.12E-02 | 6.74E-04 | 0.1803 | 0.68 |
| Lumbar | 18 | 50 | 2.7 | ASGA0080066 | 50072344 | 9.72E-03 | 5.19E-04 | 0.1428 | 0.223 |
| Thoracic/Rib | 18 | 50 | 17.35 | ASGA0080066 | 50072344 | -8.24E-02 | 2.38E-03 | 0.8261 | 0.223 |
| Thoracic/Rib | 18 | 51 | 2.5 | M1GA0023299 | 51227632 | 2.94E-02 | 1.22E-03 | 0.367 | 0.883 |
| Thoracic/Rib | 18 | 54 | 2.8 | H3GA0051131 | 54138712 | 3.31E-02 | 1.46E-03 | 0.432 | 0.872 |
| Kyphosis | X | 5 | 2.27 | MARC0074494 | 5496341 | 2.58E-02 | 1.79E-03 | 0.252 | 0.659 |
| Kyphosis | X | 39 | 3.47 | ASGA0081055 | 39658724 | -4.12E-02 | 3.21E-03 | 0.4541 | 0.56 |
| Thoracic/Rib | X | 41 | 4.72 | ASGA0081099 | 41189836 | -3.26E-02 | 1.67E-03 | 0.5191 | 0.28 |
| Lumbar | X | 117 | 0.48 | INRA0057031 | 117611622 | -2.77E-03 | 1.77E-04 | 0.0525 | 0.61 |
| Lumbar | X | 120 | 0.92 | CASI0005068 | 120937826 | 4.23E-03 | 2.66E-04 | 0.0758 | 0.642 |
| Kyphosis | X | 136 | 5.45 | ASGA0081547 | 136433302 | 8.21E-02 | 5.34E-03 | 0.7428 | 0.26 |
| Thoracic/Rib | X | 140 | 1.58 | MARC0001310 | 140124323 | -1.29E-02 | 5.11E-04 | 0.1457 | 0.928 |
