# Supplementary material for: A study of vertebra number in pigs confirms the association of vertnin and reveals additional QTL
Source: BMC Genet. 2015 Oct 30;16:129. doi: 10.1186/s12863-015-0286-9 (PMC4628235; doi:10.1186/s12863-015-0286-9)

**Additional file 2: Figure S1** Linkage disequilibrium plot for SNP markers on chromosome 7 between 103 and 105 Mb. Figure created with Haploview 4.2 (http://www.broadinstitute.org/haploview/haploview).


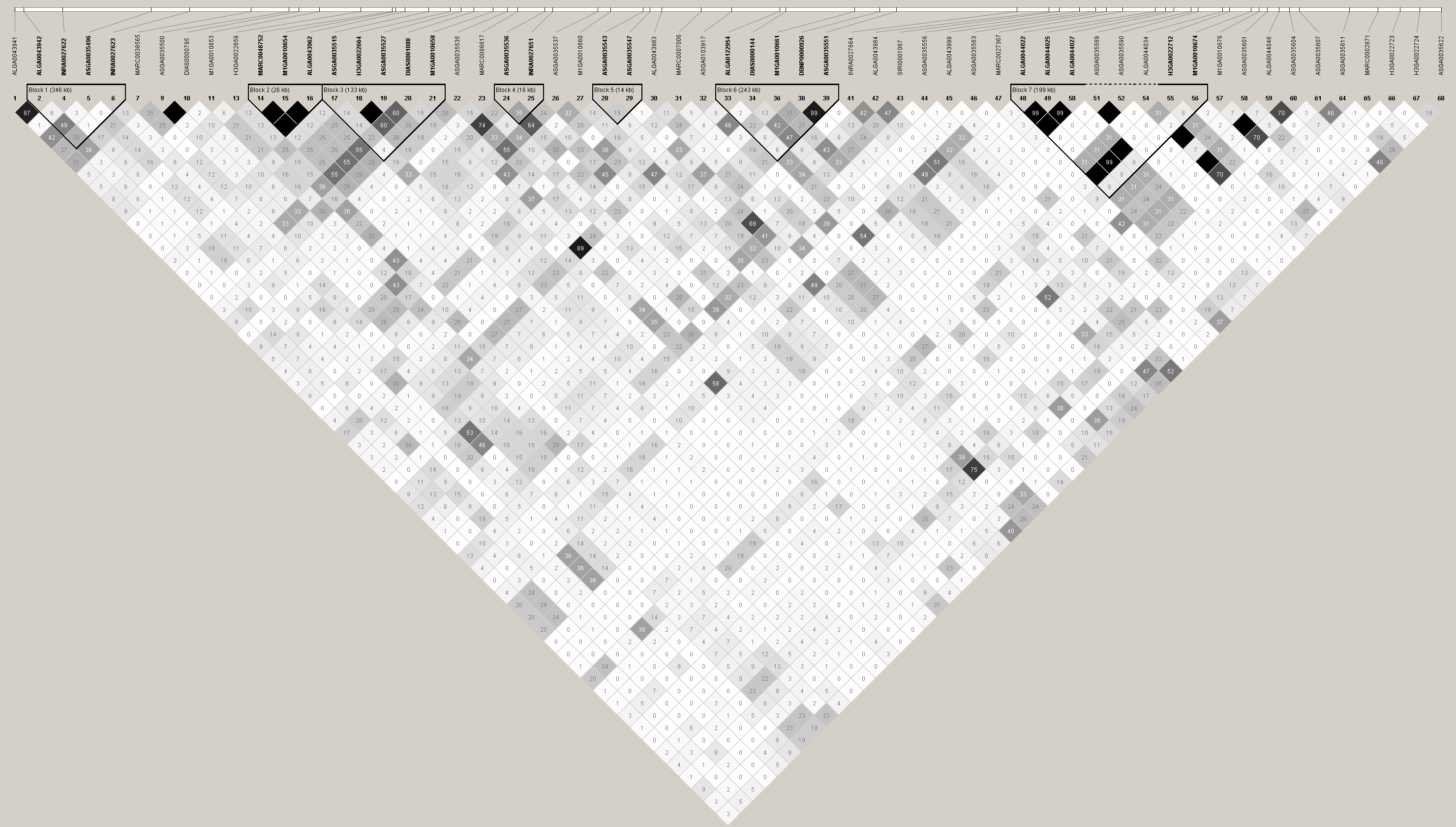

Supplement: Additional file 2: Figure S1. — Linkage disequilibrium plot for SNP markers on chromosome 7 between 103 and 105 Mb. Figure created with Haploview 4.2 (http://www.broadinstitute.org/haploview/haploview). (DOCX 359 kb) [file 12863_2015_286_MOESM2_ESM.docx]
